# Two years of gender identity service for minors: overrepresentation of natal girls with severe problems in adolescent development

**DOI:** 10.1186/s13034-015-0042-y

**Published:** 2015-04-09

**Authors:** Riittakerttu Kaltiala-Heino, Maria Sumia, Marja Työläjärvi, Nina Lindberg

**Affiliations:** University of Tampere, School of Medicine, 33014 University of Tampere, Tampere, Finland; Department of Adolescent Psychiatry, Tampere University Hospital, 33380 Pitkäniemi, Finland; Faculty of Medicine, University of Helsinki, BOX 33, 00014 Helsinki University, Helsinki, Finland; Forensic Psychiatry, Helsinki University Hospital, Helsinki, Finland

**Keywords:** Transsexualism, Gender dysphoria, Sex reassignment, Adolescent development

## Abstract

**Background:**

Increasing numbers of adolescents present in adolescent gender identity services, desiring sex reassignment (SR). The aim of this study is to describe the adolescent applicants for legal and medical sex reassignment during the first two years of adolescent gender identity team in Finland, in terms of sociodemographic, psychiatric and gender identity related factors and adolescent development.

**Methods:**

Structured quantitative retrospective chart review and qualitative analysis of case files of all adolescent SR applicants who entered the assessment by the end of 2013.

**Results:**

The number of referrals exceeded expectations in light of epidemiological knowledge. Natal girls were markedly overrepresented among applicants. Severe psychopathology preceding onset of gender dysphoria was common. Autism spectrum problems were very common.

**Conclusion:**

The findings do not fit the commonly accepted image of a gender dysphoric minor. Treatment guidelines need to consider gender dysphoria in minors in the context of severe psychopathology and developmental difficulties.

## Introduction

According to the ICD-10 [1], transsexualism involves a desire to live and be accepted as a member of the opposite sex, usually accompanied by the wish to make one’s body as congruent as possible with one’s preferred sex through surgery and hormonal treatment. The desire has to be persistent and not a symptom of a mental disorder. Gender dysphoria refers to dysphoria experienced due to the incongruence between a person’s inner perception of her/his gender, and the incongruous bodily reality. The term Gender Dysphoria has also recently been adopted as the diagnostic category in DSM-5 [2]. Psychotherapeutic approaches have not proven successful in relieving gender dysphoria, and social, juridical, medical and surgical sex reassignment (SR) is nowadays the treatment of choice [3]. Sex reassignment with hormonal and/or surgical treatments has been reported to improve social, psychological and sexual well-being and functioning.

Surveys based on the Child Behaviour Checklist [4] report that 2-5% of children aged up to seven, as reported by their parents, “behaves like opposite sex” and 1-2% “wishes to be of opposite sex”, but cultural issues likely play a major role in whether a child’s behavior is perceived as gender atypical. Consultations due to gender identity are generally more often sought for boys than girls, which may suggest greater gender variation in boys, but also that effeminate behaviours in boys are perceived as more of a problem than tom-boyishness in girls [5,6].

Of children with even severe gender dysphoria and cross-sex identification, about 85% do not develop a persistent transsexual identity in adolescence (reviewed in [7]). Reliable indicators are not so far available regarding which gender dysphoric children cease to be so in puberty and who develop transsexual identity [8]. Medical interventions are therefore not warranted in pre-pubertal children. In light of current knowledge, transsexual identity in adolescence is persistent and medical interventions may be appropriate. According to the treatment model developed in the Netherlands (Dutch model), early treatment may include delaying puberty after its first stages with GnRh analogues, and administering cross-sex hormones from about age 16 [9,10]. This approach is recommended when childhood gender dysphoria exacerbates in puberty, no (primary) severe psychopathology is present, and the young person has appropriate developmental support and support for the process from her/his primary caregivers (parents). The rationale with GnRh analogue treatment is to prevent the undesired development of secondary sex characteristics and thereby facilitating later transition to the desired role, and postponing complicated and irreversible treatment decisions to a more mature age. Psychopathology largely attributed secondary to gender dysphoria is expected to be relieved by puberty blocking and resolved by sex reassignment [5,11,12].

In the past decade, the numbers of referrals to child and adolescent gender identity services have been on the increase across Europe (personal communications in 2013 and 2014 from UK, NL, Spain, Sweden child and adolescent gender identity teams) and in Canada [13]. It is not known whether this represents a true increase in gender dysphoria, lowered thresholds for seeking help for it or perhaps cultural developments that promote the conceptualization of developmental challenges as being rooted in sex and gender.

In Finland, the legislation stipulates that a transsexual person may be recognized in law as a member of the desired sex and have access to hormonal and surgical sex reassignment (in public health care) (act 2002/563). A psychiatric assessment by a specialized gender identity team is a prerequisite for legal as well as surgical sex reassignment, both of which have a lower age limit of 18. The specialized psychiatric assessment by a gender identity team is centralized to two university hospital psychiatric clinics, Tampere and Helsinki University Hospitals, in the country (codes 1053/2002 and 476/2010).

Since 2011, specialized adolescent psychiatric gender identity teams have been available for minors at the above mentioned two university hospitals. The excessive number of referrals, exceptional sex ratio and severity of general psychopathology among the applicants compared to what might have been anticipated on the basis of the literature called for clinical attention from the beginning of the service. The aim of this study is to describe the adolescent applicants for legal and medical sex reassignment in terms of sociodemographic, psychiatric and gender identity related factors and adolescent development in order to initiate a scientific discussion on the meaning of these observations.

## Materials and methods

The study comprises a retrospective chart review of all the SR applicants attending for assessment by one of the two adolescent gender identity services in Finland (Tampere University Hospital, Department of Adolescent Psychiatry) in 2011–2013. Altogether 49 adolescents were referred to assessment for sex reassignment and invited to their first meeting during the study periods, but two adolescents declined to start the evaluation. Thus 47 adolescents are included in this study. Of these, one was mutistic and did not provide any information; for this young person, information on personal experiences is missing but information from case records and parents could be used.

The assessments take place in an outpatient setting and comprise structured and free format assessments and interviews with an adolescent psychiatrist, a psychiatric nurse, a social worker and a psychologist. The adolescent and her/his parents/guardians are seen together and separately by all the multi-disciplinary team members. Psychiatric and medical files are requested from all previous health care contacts of the adolescent, with due permission from her/him and her/his parents. After completing all the assessments, the multi-disciplinary team discusses the diagnosis as to gender identity and mental disorders, eligibility for hormonal SR treatments and possible other needs to be met and recommendations to be given regarding gender identity needs and mental health needs when appropriate. All the below described measures were collected using all the material available after the assessment. The study received approval from the ethics committee of Pirkanmaa Hospital District.

### Measures

Sociodemographic variables collected were age, natal sex, family structure (living with both parents/one parent/neither parent) and parental education (professional/intermediate/skilled non-manual/skilled manual/unskilled or not in employment). Further background information included the reason for referral (sex reassignment, definite wish/sex reassignment, possible reassignment/other) and parental homosexuality or transsexualism (yes/no).

Throughout the discussion of our own research we use the terms “gender dysphoria” and “gender dysphoric” to refer to the experienced gender incongruence among our applicants, regardless of whether they fulfill the diagnostic criteria for Gender Dysphoria in the DSM-5. For the present study we recorded whether there had been signs of gender dysphoria/gender incongruence in childhood (before age 12) (Table 1). Age of onset of conscious gender concerns and age when the applicant was convinced that s/he is transsexual were recorded. If the adolescent was already living in the desired role (Table 1), it was recorded for how long.

**Table 1:** **Variable descriptions for childhood gender dysphoria, bullying and social isolation**

**Gender dysphoria/gender incongruence in childhood (<12 years of age)**	
Childhood gender dysphoria/incongruence present	Childhood gender dysphoria/incongruence not present
• explicit gender dysphoria or marked and persistent cross-gender identification on behavioural level even without explicit verbalization of one’s gender related thoughts and feelings in childhood	• no signs of gender dysphoria/incongruence in childhood
**Gender presentation, living in desired role**	
Classified as living in the desired role	Not classified as living in the desired role
• the applicant had officially changed her/his name to a gender neutral one or arranged his/her registration in school, and being consistently called, by a name suggesting the desired sex; being always presented to new people as being of the desired sex; being treated by family, teachers/employers, friends, schoolmates as well as by new people as a member of the desired sex	• the adolescent had not made any attempt to live and be treated in the desired role
• some of these young people had explicitly “come out” in school and openly made a transition to the desired role; some, with the support of some community key adults, had adults, had totally concealed their natal sex from the school/workplace	• the adolescent dressed gender neutrally and asked the family to use a name indicative of the desired sex, but was actually not living in any social role outside the family due to isolation from social interactions
	• some of the adolescents in this group were almost totally isolated in their homes (not going to school or work, not meeting peers), some attended school but were isolated from social interactions there and elsewhere
**Bullying**	
Significantly subjected to bullying	Not subjected to bullying
• the applicant and/or her/his parents considered that there had been significant and traumatic victimization.	• no recollection of being bullied
• a) related to gender presentation or sexual identity: name-calling, spreading rumours and the like related to gender presentation/sexual identity	• if ever bullied, the adolescent described it as non-significant (“maybe sometimes”, “not more than anyone else”).
• b) not related to gender or sexual identity: bullying was related to other issues like weight, interests, belonging or not belonging to a certain group etc.	
**Isolation**	
Periods of isolation	No isolation
• periods of not having contact with peers outside of arranged study-related activities at school - or not even that, if not attending school	• no interruptions in attending age appropriate daily programme (usually school), having age-appropriate contacts with peers
• having same-age contacts only with one’s own siblings	
• keeping (tenuous, infrequent) contact with one or two peers only despite previously having been normatively engaged with peers	
• contacts outside the family only via Internet	

Previous and current psychiatric history was recorded. Previous files were not always complete, and diagnoses were not always accurately defined in terms of ICD-10 diagnostic codes. Thus, we recorded 1) whether the young person had been in contact with psychiatric services prior to entering the gender identity service (yes/no), 2) whether the previous contact had been because of gender concerns or psychiatric symptoms (gender issues only/psychiatric symptoms only/both), 3) what kind of problems the young person had displayed (anxiety, depression, suicidal behaviours, conduct problems, autism spectrum related problems, substance abuse, psychotic symptoms, other; all recorded yes/no), and 4) the temporal relationship between psychiatric symptoms and gender dysphoria/identity concerns (psychiatric symptoms emerged earlier/gender dysphoria and gender identity concerns emerged first).

Peer-related difficulties were recorded being subjected to bullying at school (yes/no) and isolation from peers (yes/no) (Table 1). Of bullying it was recorded whether it happened before, after or both before and after of the onset of gender dysphoria, and whether it was related to gender presentation or sexual orientation. Of social isolation it was recorded whether it occurred before, after or both before and after the onset of gender dysphoria.

### Statistical analyses

All the variables were recorded in a structured form developed for this research. Descriptive analysis was conducted using statistical methods for quantitative data. We report frequencies and means (sd) where appropriate. Between groups comparisons are made with cross-tabulations and chi-square statistics/Fisher’s exact test, and with t-test where appropriate.

### Qualitative observations

The qualitative content analysis approach [14] was applied to illustrate, based on all material recorded in case histories, different groups of gender dysphoric adolescents, or different developmental pathways resulting in the adolescent now perceiving the need to apply for sex reassignment. This was carried out by condensing and extracting from all material recorded in the case histories similar and different developmental patterns and descriptions of experiences that could be used to create mutually exclusive model stories, or trajectories that would include all the studied adolescents and not allow for assigning a given adolescent to more than one trajectory. The model stories were not defined in advance but they were formed in a data-driven process, the outcome of which is presented.

## Results

### Demographics

Of the applicants included in the present study, 41 were natal girls and 6 were natal boys. Their mean age (sd) at entering assessment was 16.04 (0.57) years for natal boys and 16.66 (1.07) for natal girls (p = 0.18). Of these, 49% (23) were living with both their biological parents, 39% (18) with one biological parent, and 13 % (6) in child welfare foster placements or independently. Parental education was distributed as follows: 16% (8) professional, 5% (2) intermediate, 22% (10) skilled non-manual, 43% (20) skilled manual, and 14% (7) were unskilled or not participating in work life. None of the applicants had transsexual or homosexual parents.

### Gender dysphoria

Of the applicants, 32% (14/47) reported having started to consciously question their gender before age 12, 62% (30/47) at 12 or later, and three applicants (6%) could not define this. Most commonly (one in five) these concerns had started at age 14. There were altogether five applicants (11%) who during childhood had persistently expressed gender dysphoria and/or identified with the opposite sex, and three (6%) who during childhood had transiently displayed gender dysphoria and a desire to be of the opposite sex. A further nine applicants (19%) had been tomboyish girls but had not questioned their gender or experienced dysphoria, and as to most of the applicants (30/47, 64%), neither the young person nor her/his parents recalled gender dysphoria or cross-gender behaviors during childhood.

During the assessment process, 72% (34/47) of the applicants were sure about feeling they were of the opposite sex to their natal and about pursuing sex reassignment, but 28% (13/47) were not sure about their feelings regarding gender identity and/or sex reassignment. There was no difference between natal girls and natal boys in this regard. Of those who felt sure about their cross-gender identity, 15% (5/34) recalled reaching the conclusion before age 12, 79% (27/34) at 12 or later, and two (6%) could not define at what age they had reached the conclusion. There was no difference between natal girls and natal boys. The time frame from first becoming aware of gender dysphoria to being sure of one’s own cross-gender identity ranged from 0 to 7 years, with mean 1.6 (sd 2.1) years.

Of all the applicants, 38% (17/47) were living in the desired role when the assessment was completed, 50% (3/6) of the natal boys and 37% (15/41) of the natal girls (p = 0.41). Of those applicants who expressed certainty about being of other than their natal sex and desiring physical and legal sex reassignment, 47% (16/34) were living in the desired role. Of those who were living in the desired role, the mean (sd)/median time of living in the role was 28.3 (17.9)/24.0 months for natal boys, and 29.8 (39.2)/12 months for natal girls (p = ns).

### Peer relationship difficulties

Of the applicants, 57% (27/47) had been significantly bullied at school, 53% (25/47) in primary school (grades 1–6, ages 7–13 yrs) and 45% (21/47) in secondary school (grades 7–9, ages 13–16 yrs). Of those who had been victims of bullying, 73% (19/27) had been bullied before they came to think about their gender identity, 8% (2/27) after starting to think about gender issues, and 19% (5/27) both before and after. Of those bullied, 27% (7/26) reported that bullying had been related to gender presentation or sexual identity, and 73% (19/26) had been bullied due to some other reasons (see Table 1).

Natal girls and natal boys had been bullied equally frequently. Natal girls tended more often to report having been bullied only before the onset of gender dysphoria, and natal boys more often both before and after the onset of gender dysphoria (girls: 78% (17/23) only before, 9% (2/23) only after, 13% (3/23) before and after vs. boys: 33% (1/3) only before, none only after, 67% (2/3) both before and after, p = 0.08). Among natal boys gender presentation and/or sexual identity had always been the topic of the bullying, among natal girls 83% (19/23) had been bullied for something else and 17% (4/23) due to gender presentation/sexual identity (p = 0.01).

Of the applicants, 45% (21/47) had presented with periods of isolation from peer relationships; 32% (15/47) before and 40% (19/47) after the onset of gender dysphoria, and 43% (20/47) were socially isolated during the SR assessment. Twenty-eight per cent (13/47) were isolated in all three observed periods. Social isolation was equally common among natal boys and girls applicants.

### Psychiatric treatment and psychopathology

Seventy-five per cent of the applicants (35/47) had been or were currently undergoing child and adolescent psychiatric treatment for reasons other than gender dysphoria when they sought referral to SR assessment, and two more were contacted with general adolescent psychiatric services soon after entering the SR assessment. Sixty-four per cent (30/47) were having or had had treatment contact due to depression, 55% (26/47) due to anxiety disorders, 53% (25/47) due to suicidal and self-harming behaviours, 13% due to psychotic symptoms (6/47), 9% (4/47) due to conduct disorders, 4% (2/47) due to substance abuse, 26% (12/47) due to autism spectrum disorder, and 11% (5/47) due to ADHD. One severe case of anorexia nervosa was noted. Of the applicants, 68% (32/47) had had their first contact with psychiatric services due to other reasons than gender identity issues. Natal boys and natal girls had equally commonly been treated for psychiatric disorders except for ADHD which had been more commonly treated in natal boys (50% vs.5%, p = 0.01). The mean number of distinct psychiatric problems was 2.3 (sd 1.7), with no difference between natal girls and natal boys.

### The different groups

Five different mutually exclusive groups (a - e below) were identified that differed as to onset of gender dysphoria and cross-gender identification, psychopathology and adjustment/difficulties in social relationships, and the temporal relationships between these. They are presented in Table 2.

**Table 2:** **The different groups of gender dysphoric adolescents seeking SR**

Early onset gender dysphoria, exacerbates in puberty	
a) with no with no significant psychopathology and developmental problems (n = 2)	b) with considerable psychopathology and developmental problems (n = 3)
• very mild or no psychopathology across childhood and until the assessment	• severe psychopathology that had previously and currently required specialist level child and adolescent psychiatric care (autism spectrum disorder, OCD, Tourette, anorexia nervosa, suspected psychotic episodes or psychosis high risk, specific learning difficulties)
Adolescent onset gender dysphoria, where transsexual identity appeared established	
c) without, or with only mild psychopathology and developmental difficulties (n = 10)	d) with severe psychopathology and developmental difficulties (n = 9)
• mild to moderate depression or anxiety, could be considered secondary to gender dysphoria, or was transient, and did not impair functioning in social relationships or school	• psychiatric problems that warranted specialist level adolescent psychiatric treatment, either in treatment at the beginning of their SR assessment, or treatment contact was arranged during the SR assessment
• age-appropriate social relationships and leisure time activities, participation in age-appropriate educational activities (comprehensive, vocational or upper secondary school)	• autism spectrum disorders (3), major depression (3), social phobia (5), substance abuse problems (1) or a history of conduct disorder and trauma (2) (several had two disorders); clearly more severe psychopathology than what was seen in group c
e) Adolescent onset gender dysphoria, identity confused development (n = 23)	
• In childhood, no gender dysphoria nor cross-gender behaviors	
• For most of their primary school years (age 7–12 years) felt excluded	
• Persistent experiences of bullying before the onset of gender dysphoria	
• In adolescence, social anxiety and depression, most often with self-harm and suicidal preoccupation if not suicide attempts	
• Isolated	
• Long periods of not attending school, or if attended school, did not engage in peer contacts outside learning situations arranged by teachers.	
• Did not meet with same-aged peers in leisure time, or they met with few peers and only if their parents arranged it; many in contact only with their family members.	
• Socially and/or academically marginalized	
• Very high expectations that SR would solve their problems in social, academic, occupational and mental health domains	

We carried out logistic regression analyses to detect what kind of presenting features were associated with belonging to the last, confused group of adolescents with gender dysphoria (e) when entered in the model simultaneously. This was appropriate because psychiatric symptoms and psychosocial functioning are strongly interrelated. Age and natal sex were not predictive of belonging to the confused group. Each psychiatric problem, being subjected to bullying, presenting with periods of isolation, number of different psychiatric problems, and months living in desired role were each in turn entered as independent variables, controlling for age and natal sex. When controlling for age and natal sex, group memberships was predicted by anxiety (OR 4.8, 95% CI 1.4-17.0), suicidality (OR 5.7, 95% CI 1.7-20.3), number of different psychiatric symptoms (OR 1.7, 95% CI 1.1-2.6), and presenting with periods of isolation (OR 9.0, 95% CI 2.3-34.7). However, when presenting with periods of social isolation was entered into any other model, the other independent variables were leveled out, suggesting that social isolation was the strongest factor predicting membership of the problematic, identity confused group.

## Discussion

The number of referrals exceeded expectations. Given the most cited epidemiological figures among adults, 1:10 000–1:30000 MtF and 1:40 000–1:100 000 FtM [6], in Finnish population, 6–18 boy-to-girl adolescents and 2–4 girl-to-boy adolescents aged 13–18 would be expected. The number of referrals to the study unit already doubled the less conservative estimates based on adult figures. Referrals to the other adolescent gender identity unit amount to equal numbers, and the natal girl:boy ratio in referrals is also similar in the other unit (Tainio V-M, personal communication). Valid epidemiological research on incidence and prevalence of transsexualism or gender dysphoria at large among adolescents is not available [6]. The adult figures cited above are based on treatment seeking, as are the numbers presented in the present study. Gender dysphoria may be more common among adolescents than among adults, or it may be increasing in younger age cohorts.

Not all applicants could be seen as presenting with established transsexual identity, even though they suffered gender dysphoria. Excluding the confused (e) group in our data, 3 boy-to-girl and 21 girl-to-boy applicants were identified who displayed transsexual identity that appeared established, unique, and not part of more general identity confusion or secondary to severe mental disorders. Given that these numbers are based on half of the adolescent gender identity assessments in Finland, the findings further suggest that severe and persistent gender dysphoria/transsexualism in adolescence may be more common than hitherto assumed.

The natal girl:boy ratio among the adolescent SR applicants was very high. In prepubertal children referred to gender identity services, boy:girl ratio is reportedly 3–6:1, with some variation across countries presumably due to cultural reasons [5,13]. Previously a more even boy:girl ratio has been suggested in adolescents seeking sex reassignment than among child samples [13], and a recent paper from Germany reported natal boy:natal girl ratio of 0.81 among 268 minors diagnosed with gender identity disorder from 1987–2013 [15]. Among adults, there seems to be remarkable variation across countries in the ratio of natal men:natal women seeking for sex reassignment [16]. In Western countries natal male transsexuals exceed natal females transsexuals. A German study demonstrated that the natal male:natal female ratio among transsexual people has changed to more equal towards 2000’s that what it was in earlier decades [16]. However, the overrepresentation of girls on our sample differs still from these more recent trends, and it is similar in both the two Finnish centers. We have so far no explanation for this great overrepresentation of natal girls seen in our material, and equalizing of sex ratio demonstrated by others [13,15,16]. Cultural trends may somehow influence this. May be more permissive societal attitudes allow “coming out” as gender variant more easily than before. However, why this would concern primarily girls remains an open question.

Of children and adolescents, 10-15% are regularly (weekly) involved in school bullying [17]. Of the adolescent SR applicants, more than a half had been subjected to bullying. Even if in the present study it was not possible to verify exactly how frequently the applicants had been bullied, we only recorded bullying that the adolescent and her/his parents perceived as significant: particularly intensive, vicious, long-term and traumatizing. However, in more than two thirds of the cases, bullying had occurred before the onset of gender dysphoria, and was not targeted at gender or sexual identity. Bullying is an unspecific risk factor for developmental problems rather than a problem specifically related to gender identity. That natal boys were more commonly bullied because of gender presentation suggests that effeminate characteristics in boys are less tolerated than masculine self-presentation in girls.

Peer relationships are of the outmost importance during adolescent development [18-20], and social isolation from peer relationships suggests developmental difficulties and impaired mental health [21-24]. In the present sample, isolation was extremely common and also the strongest predictor of membership of the “confused” group.

More than three quarters of the adolescent SR applicants had needed and/or currently needed specialist level child and adolescent psychiatric services due to psychiatric problems other than gender dysphoria. Specialist level child and adolescent psychiatric services are provided exclusively for severe disorders in Finland [25,26]. The recorded comorbid disorders were thus severe and could seldom be considered secondary to gender dysphoria. This utterly contradicts the findings in the Dutch child and adolescent gender identity service, where two thirds of adolescent SR applicants did not have psychiatric comorbidity [27]. In a recent German study, 43% of children and adolescents seen in a specific gender identity unit suffered from major psychopathology [15]. For the time being, we are unable to explain why Finnish adolescent SR applicants appear psychiatrically much more disturbed than has been reported elsewhere, but our findings warrant attention. The treatment guidelines for adolescent gender dysphoria may require extensions taking into account the needs of those with severe psychopathology and identity confusion, very unlikely currently eligible to medical SR.

The overlap between autism spectrum disorders and gender dysphoria has been recognized before [28]. In a Dutch gender identity service, 9.4% of adolescents presented with autism spectrum disorder. In our sample, 26% of the adolescent SR applicants were diagnosed to be on the autism spectrum. These diagnoses had mainly been made during the adolescents’ previous psychiatric treatment in our hospital or elsewhere, but three such diagnoses were also made by our team. In our hospital, the ADOS [29] is used with the minors, and the 3Di [30] or ADI-R [31] with parents to diagnose autism spectrum disorders. We could not systematically review with which protocols the diagnoses had been made elsewhere in the country, but in our clinical opinion there was no reason to doubt them. It is currently not known why autism spectrum is overrepresented in gender dysphoric children and adolescents. The conditions could be truly co-occurring. Prenatal exposure to high levels of testosterone could be involved in the development of both conditions, especially for girls with autism spectrum disorder, but this leaves the comorbidity in males unexplained. Gender identity issues could arise from autism spectrum people’s predisposition toward unusual interests, or gender dysphoria in ASD could represent OCD rather than genuine gender identity issues. The cross-gender behaviour in ASD minors could also rather represent non-normative sexual interests or unusual sensory preferences [28]. Our clinical impression is that a long-standing feeling of being different and an outsider among peers could play a role in ASD children developing gender dysphoria in adolescence. In our clinical sample of gender dysphoric adolescents, autism spectrum disorders by far exceeded the prevalence of 6/1000 suggested for general population [32], and almost three-fold that in the sample of deVries et al. [28]. Autism spectrum needs to be taken seriously in considering treatment guidelines for child and adolescent gender dysphoria. Given the nature of ASD, particularly ASD children’s and adolescents’ difficulties in adjusting to changes, profound changes in their own bodies with SR treatments may pose a major challenge to psychological adjustment, and ASD adolescents may be particularly rigidly unwilling to consider this in advance.

In the international literature on gender dysphoria in minors, the most often portrayed picture is that of childhood cross-gender identification/gender dysphoria, where gender dysphoria exacerbates in puberty due to the development of secondary sex characteristics. Our findings suggest that there are many more developmental pathways that may also need different treatment approaches. In our data, most of the adolescents first presented with gender dysphoria and cross-gender identification well after the onset of puberty, and the vast majority suffered significant psychopathology and broader identity confusion than gender identity issues alone. It is important to be able to openly discuss these alternative presentations of gender dysphoria in order to find appropriate treatment options.

Adolescence is a period of identity formation. From early to late adolescence, identity develops from fragmented and contextual identity experience to endogenous, permanent and integral identity that remains constant across contexts and interactions [33]. Identity is formed through diverse physical and psychological developments and in relation to other people and the social environment [34,35]. An adolescent also faces fundamental identity challenges in the domains of religion, worldview, ethnicity, sexuality and the like. Identification with various groups is often passionate during adolescence, but the object of identification may also change, even several times [34-37]. Adolescents are more suggestible and submit more readily to group pressure to gain acceptance [38]. Adolescence is a period of maturation of social cognition, and a prerequisite for the maturation of social cognition is the maturation of the central nervous system that continues to the third decade of life [39]. During puberty and adolescent development there may be some overlap between normative testing of sexuality and gender roles in the one end, and gender dysphoria as a disorder in the other end of the spectrum. This would implicate that GD in adults and in adolescence may not be the same issue in general. For these reasons it is more challenging to assess whether the gender identity of an adolescent is so firmly established that physical intervention is indicated than it is to assess this among adults.

In the majority of the applicants, gender dysphoria presented in the context of wider identity confusion, severe psychopathology and considerable challenges in the adolescent development. At this point it is not possible to predict how gender dysphoria in this group will develop: will gender dysphoria in these adolescents cease with the resolution of wider developmental problems, or perhaps consolidate later into transsexual identity, with the completion of the developmental tasks of adolescence.

### Methodological considerations

The present paper is based on information on all adolescents who entered the assessment for sex reassignment in Finland in 2011–2013 by one of the two centralized adolescent gender identity teams in the country. The basis for choosing one or another of the two centers was geographical and not likely to create bias due to subject selection. It is further known that number of referrals during the study period well as natal girl:natal boy ratio are similar in both centers.

The data collection was systematic and structured, which adds to the reliability of the findings. The data collection took place in the form of retrospective chart review of files created during a comprehensive assessment period by a multi-disciplinary team. Thus data collection was unlikely to bias the assessments in any way. Comprehensive assessments by a multi-disciplinary team are likely to provide reliable and valid data. The multi-disciplinary team collected information from the applicants themselves, from their parents, from previous case histories and by their own psychometric measurements. The applicants themselves might be prone to interpret a variety of their problems as being a result of gender incongruence, even if the problems actually were independent of gender identity issues or even predisposing to gender incongruence. In this study we attempted to avoid bias due to subjects’ interpretation by using multiple source of information.

However, the data is relatively small and does not permit complex analyses. The study remains descriptive and cannot shed light on causal relationships. Some information of interest for the research was occasionally missing in the files, because the files were primarily created for clinical purposes, not for research.

The validity of the diagnoses in previous psychiatric contacts needs to be considered with certain caution. Previous files were not always complete and did not provide diagnoses according to ICD-10, and we were not able to check in the databases of the previous treatment providers what ICD diagnoses were recorded there. Thus, we recorded reasons for previous treatment based on verbalizations in the referrals and available copies of previous files. This only allowed a rough descriptive classification to problems related to anxiety, depression, suicidal behaviours, conduct problems, autism spectrum related problems, substance abuse, psychotic symptoms and other. We only recorded these problems if the adolescent had had a psychiatric treatment contact. The data gives a picture of the primary problems in previous psychiatric treatment contacts but not of all possible symptoms. Thus, our figures for problems related to anxiety, depression etc. are likely underestimates. It was also not possible to obtain exact information of when the various symptoms and disorders had been present and for how long time, except for autism which is of course assumed a lifetime condition. However, as clinical research on adolescent SR applicants is scarce, descriptive studies are valuable in providing a basis for discussion and international comparisons that are needed in order to create optimal clinical treatment guidelines.

Psychotic symptoms in our data mainly comprise brief and limited hallucinatory experiences. Psychotic symptoms were recorded if there were descriptions of hallucinations in the files, or of the previous files mentioned “psychotic symptoms” even when not giving more detailed descriptions. However, none of the applicants had a diagnosis of schizophrenia or schizoaffective disorder. Assessing gender dysphoria in the context of schizophrenia spectrum psychoses would be inappropriate. Doctors/units primarily contacted would very unlikely refer a patient with schizophrenia or schizoaffective disorder in gender identity assessments. Current psychotic symptoms would result in the gender identity team promptly referring the young person to general adolescent psychiatric care.

The findings cannot be generalized to all adolescents experiencing gender variation. Not all gender incongruent people perceive a need to seek for SR, or find it timely during adolescence.

## Conclusion

Adolescents seeking sex reassignment represent a variety of developmental pathways differentiated by the timing of onset of gender dysphoria, psychopathology and developmental difficulties. It is important to be aware of the different groups, or developmental pathways, in gender dysphoric adolescents in order to be able to find appropriate treatment options. In the presence of severe psychopathology and developmental difficulties, medical SR treatments may not be currently advisable. Treatment guidelines need to be reviewed extended to appreciate the complex situations.

## References

[1] World Health Association (1992). International Statistical Classification of Diseases and Related Health Problems, 10th revision (ICD-10).

[2] American Psychiatric Association (2013). Diagnostic and Statistical Manual of Mental Disorders, Fifth edition DSM-5).

[3] Coleman E, Bockting W, Botzer M, Cohen-Kettenis P, DeCuypere G, Feldman J (2011). The Standards of Care of the World Professional Association for Transgender Health, 7th version. Int J Transgenderism.

[4] Achenbach TM, Edelbrock C (1983). Manual for the Child Behavior Checklist and Revised Child Behavior Profile.

[5] Möller B, Schreier H, Li A, Romer G (2009). Gender identity disorder in children and adolescents. Curr Probl Pediatr Adolesc Health Care.

[6] Zucker KJ, Lawrence AA (2009). Epidemiology of gender identity disorder. Int J Transgenderism.

[7] Steensma T (2013). From gender variance to gender dysphoria. Psychosexual development of gender atypical children and adolescents. Academic dissertation, Vrije Universiteit Amsterdam.

[8] Steensma DT, Biemond R, deBoer F, Cohen-Kettenis PT (2011). Desisting and persisting gender dysphoria after childhood: a qualitative follow-up study. Clin Child Psychol Psychiatry.

[9] Cohen-Kettenis P, Steensma T, deVries AL (2012). Treatment of adolescents with gender dysphoria in the Netherlands. Child Adol Psychiatric Clinics N Am.

[10] deVries AL, Cohen-Kettenis P (2012). Clinical management of gender dysphoria in children and adolescents. The Dutch approach. J Homosexuality.

[11] Wren B (2000). Early physical intervention for young people with atypical gender identity development. Clin Child Psychol Psychiatry.

[12] deVries A, Cohen-Kettenis P, Delemarre-van de Maal H (2006). Caring for transgender adolescents in BC: suggested guidelines. Clinical management of gender dysphoria in adolescents.

[13] Wood H, Sasaki S, Bradley S, Singh D, Fantus S, Owen-Anderson A (2013). Patterns of referral to a gender identity service for children and adolescents (1976–2011): age, sex ratio, and sexual orientation. J Sex Marital Ther.

[14] Graneheim U, Lundman B (2004). Qualitative content analysis in nursing research: concepts, procedures and measures to achieve trustworthiness. Nurse Educ Today.

[15] Meyenburg B (2014). Geschlechtsdysphorie im Jugendalter. Schwierige Behandlungsverläufe. Prax Kinderpsychol Kinderpsychiat.

[16] Garrels L, Kockott G, Michael N, Preuss W, Renter K, Schmidt G (2000). Sex ratio of transsexuals in Germany: the development over three decades. Acta Psychiatr Scand.

[17] Kaltiala-Heino R, Fröjd S (2011). Correlation between bullying and clinical depression in adolescent patients. Adolescent Health Med Therapeutics.

[18] Larson R, Richards MH (1991). Daily companionship in late childhood and early adolescence. Changing developmental contexts. Child Dev.

[19] Hall-Lande J, Eisenberg M, Christenson S, Neumark-Sztainer D (2007). Social isolation, psychological health, and protective factors in adolescence. Adolescence.

[20] Laursen B, Hartl A (2013). Understanding loneliness during adolescence: developmental changes that increase the risk of perceived social isolation. J Adolescence.

[21] Rubin KH, Root AK, Bowker J, Gazelle H, Rubin KH (2010). Parents, peers, and social withdrawal in childhood: a relationship perspective. Social anxiety in childhood: bridging developmental and clinical perspectives. New Directions for Child and Adolescent Development, 127, 79–94.

[22] Shevlin M, Murphy S, Mallett J, Stringer M, Murphy J (2013). Adolescent loneliness and psychiatric morbidity in Northern Ireland. Br J Clin Psychol.

[23] Harris R, Qualter P, Robinson S (2013). Loneliness trajectories from middle childhood to pre-adolescence: impact on perceived health and sleep disturbance. J Adolescence.

[24] Matheson S, Vijayan H, Dickson H, Shepherd A, Carr V, Laurens K (2013). Systematic meta-analysis of childhood social withdrawal in schizophrenia, and comparison with data from at-risk children aged 9–14 years. J Psychiatr Res.

[25] Kaltiala-Heino R, Fröjd S, Autio V, Laukkanen E, Närhi P, Rantanen P (2007). Transparent criteria for specialist level adolescent psychiatric care. Eur Child Adolesc Psychiatr.

[26] Isojoki I, Fröjd S, Rantanen P, Laukkanen E, Närhi P, Kaltiala-Heino R (2008). Priority criteria tool for elective specialist level adolescent psychiatric care predicts treatment received. Eur Child Adolesc Psychiatr.

[27] deVries AL, Doreleijers TA, Steensma TD, Cohen-Kettenis PT (2011). Psychiatric comorbidity in gender dysphoric adolescents. J Child Psychol Psychiatry.

[28] deVries A, Noens I, Cohen-Kettenis P, van Berckelaer-Onnes I, Doreleijers T (2010). Autism spectrum disorders in gender dyspohoric children and adolescents. J Autism Dev Disord.

[29] Lord C, Rutter M, DiLavore PS, Risi S (1999). Autism Diagnostic Observation Schedule (ADOS).

[30] Skuse D, Warrington R, Bishop D, Chowdhury U, Lau J, Mandy W (2004). The developmental, dimensional and diagnostic interview (3Di): a novel computerized assessment for autism spectrum disorders. J Am Acad Child Adolesc Psychiatry.

[31] Rutter M, Le Couteur A, Lord C (2003). Autism diagnostic interview-revised.

[32] Fombonne E (2005). Epidemiology of autistic disorder and other pervasive developmental disorders. J Clin Psychiatr.

[33] Harter S (1999). The construction of self.

[34] Erikson E (1965). Childhood and society.

[35] Moshman D (2011). Adolescent rationality and development. Cognition, morality, and identity. Third Edition.

[36] Savin-Williams RC, Ream GL (2007). Prevalence and stability of sexual orientation components during adolescence and young adulthood. Arch Sex Behav.

[37] Muuss RE (1965). Theories of adolescence (6th edition).

[38] Gardner M, Steinberg L (2005). Peer influence on risk taking, risk preference, and risky decision making in adolescence and adulthood: an experimental study. Dev Psychol.

[39] Sebastian C, Burnett S, Blakemore S-J (2008). Development of the self-concept during adolescence. Trends Cognit Sc.

